# Inducing and monitoring photothrombotic stroke in anesthetic neuroprotection-free mice using functional photoacoustic microscopy

**DOI:** 10.1117/1.NPh.13.2.025007

**Published:** 2026-04-15

**Authors:** Congsen Li, Kexin Chen, HaiPing Cai, Xiaobin Hong, Jiangbo Chen

**Affiliations:** a South China University of Technology, School of Mechanical and Automotive Engineering, Guangzhou, China; b Guangdong Provincial People’s Hospital (Guangdong Academy of Medical Sciences), Southern Medical University, Department of Neurosurgery, Guangzhou, China

**Keywords:** functional photoacoustic microscopy, photothrombotic stroke, awake mouse model, anesthetic neuroprotection, cerebral microvessels

## Abstract

**Significance:**

Preclinical ischemic stroke models frequently rely on inhalational anesthetics, which can alter cerebrovascular dynamics and confound ischemic outcomes. The specific impact of anesthesia at the microvascular level remains poorly defined, limiting the translational relevance of preclinical findings.

**Aim:**

We aim to utilize an optical resolution photoacoustic microscopy (OR-PAM) system for targeted photothrombosis (PT) induction and functional imaging and compare ischemic responses between awake and anesthetized mice to avoid anesthesia-induced confounding effects.

**Approach:**

We employed an OR-PAM system integrated with vascular stimulation capabilities, enabling PT induction in specific vessels and functional imaging of cortical brain regions. By combining with a head-restrained apparatus and photochemical thrombosis, we compared oxygen saturation (${\mathrm{sO}}_{2}$), oxygen extraction fraction (OEF), and vascular morphology [arteriovenous diameter, total vessel length (TVL), branch index (BI), vessel area density (VAD)] between awake and anesthetized mice. Behavioral tests and pathological examination were conducted to validate cerebrovascular outcomes.

**Results:**

Before PT, awake mice exhibited narrower arteriovenous diameter and higher OEF compared with anesthetized mice induced by anesthesia. Following PT, awake mice displayed more severe acute ${\mathrm{sO}}_{2}$ reduction, greater chronic vascular structure degeneration, and more delayed recovery among TVL, BI, and VAD, compared with anesthetized mice. These cerebrovascular outcomes of photothrombotic stroke in anesthetic neuroprotection-free awake mice were further validated by behavioral tests and pathological examination.

**Conclusions:**

Our findings address the critical gap of microvascular-level analysis in anesthesia-confounded stroke models. The proposed approach provides a reliable and reproducible tool for the precise induction of cerebral ischemia and dynamic monitoring of vascular changes.

## 1 Introduction

Ischemic stroke is the leading cause of death and long-term disability globally, imposing a heavy socioeconomic burden.^1^ Preclinical research relies heavily on rodent models to explore stroke pathophysiology and evaluate therapies. Middle cerebral artery occlusion (MCAO) and photothrombosis (PT) are two widely utilized experimental models for inducing ischemic stroke.^2^^–^^4^ Inhaled anesthetics, such as isoflurane, are often implemented in both MCAO and PT models. Nevertheless, accumulating evidence shows that anesthesia exerts neuroprotective effects, including vasodilation, inflammatory suppression, and reduced cerebral metabolism, which may mask intrinsic ischemic responses, including neurovascular dynamics and behavioral alteration.^5^^–^^7^

Microvessels (arterioles, capillaries, and venules) constitute the functional core of the neurovascular unit. Their structural integrity and metabolic dynamics directly govern the extent of ischemic injury and recovery potential, making them a pivotal target in stroke pathophysiology research.^8^^,^^9^ During ischemic stroke, microvessels undergo distinct pathological alterations, including rarefaction, dysregulated oxygen extraction, and increased permeability. These changes not only exacerbate local tissue hypoxia and necrosis but also impede collateral circulation establishment.^10^^,^^11^ Compared with MCAO, PT has gained increasing preference in reliable modeling due to its high reproducibility in generating ischemic lesions with minimal invasiveness and low mortality. However, conventional PT models rely on imprecise targeting and nonselective illumination, failing to precisely localize single microvessel subtypes and resulting in heterogeneous infarct regions, whereas the ill-defined irradiation site makes it difficult to control infarct extent and avoid collateral damage to normal microvasculature. Consequently, the specific impact of anesthesia on cerebral microvascular structure and function during stroke remains incompletely understood.

Various optical imaging modalities have been used for neurovascular study. Laser speckle contrast imaging (LSCI) supports quantitative assessment of cerebral blood flow (CBF) in stroke research, yet its sensitivity toward microvascular morphology remains an inherent challenge.^12^ Optical coherence tomography (OCT) excels at delivering high-resolution structural imaging, and it faces certain limitations when it comes to the direct measurement of oxygen saturation (${\mathrm{sO}}_{2}$).^13^ Fluorescence imaging (FLI) enables highly specific visualization of targeted structures or molecules with cellular-level resolution via exogenous probes but is limited by shallow penetration depth, phototoxicity, and reliance on external labeling.^14^ These imaging modalities cannot directly or noninvasively characterize the oxygen metabolism-related parameters. Optical resolution photoacoustic microscopy (OR-PAM) leverages the photoacoustic effect to realize label-free *in vivo* imaging with micron-sized precision and millimeter penetration depth,^15^^,^^16^ showing unique technical advantages in characterizing the cerebral vessels.^17^^–^^19^ Although these imaging modalities provide viable approaches for visualizing the PT model, they traditionally require separate systems for ischemia induction and imaging, thereby increasing experimental costs and compromising research efficiency.

To efficiently reveal the effect of anesthesia on photothrombotic stroke at the microvascular level, we present a functional OR-PAM-based approach integrated with PT and a head-restrained apparatus, enabling targeted induction of cerebral vascular infarction and monitoring in awake mice. This method eliminates anesthetic confounding effects, achieves vessel-specific focal ischemia induction, and realizes longitudinal quantification of vascular structure and function throughout stroke progression. This study aims to offer a physiologically relevant preclinical stroke platform, improve clinical translation of rodent studies, and facilitate neurovascular pathophysiology research without anesthetic interference.

## 2 Materials and Methods

### 2.1 Imaging System Setup

**Fig. 1:**
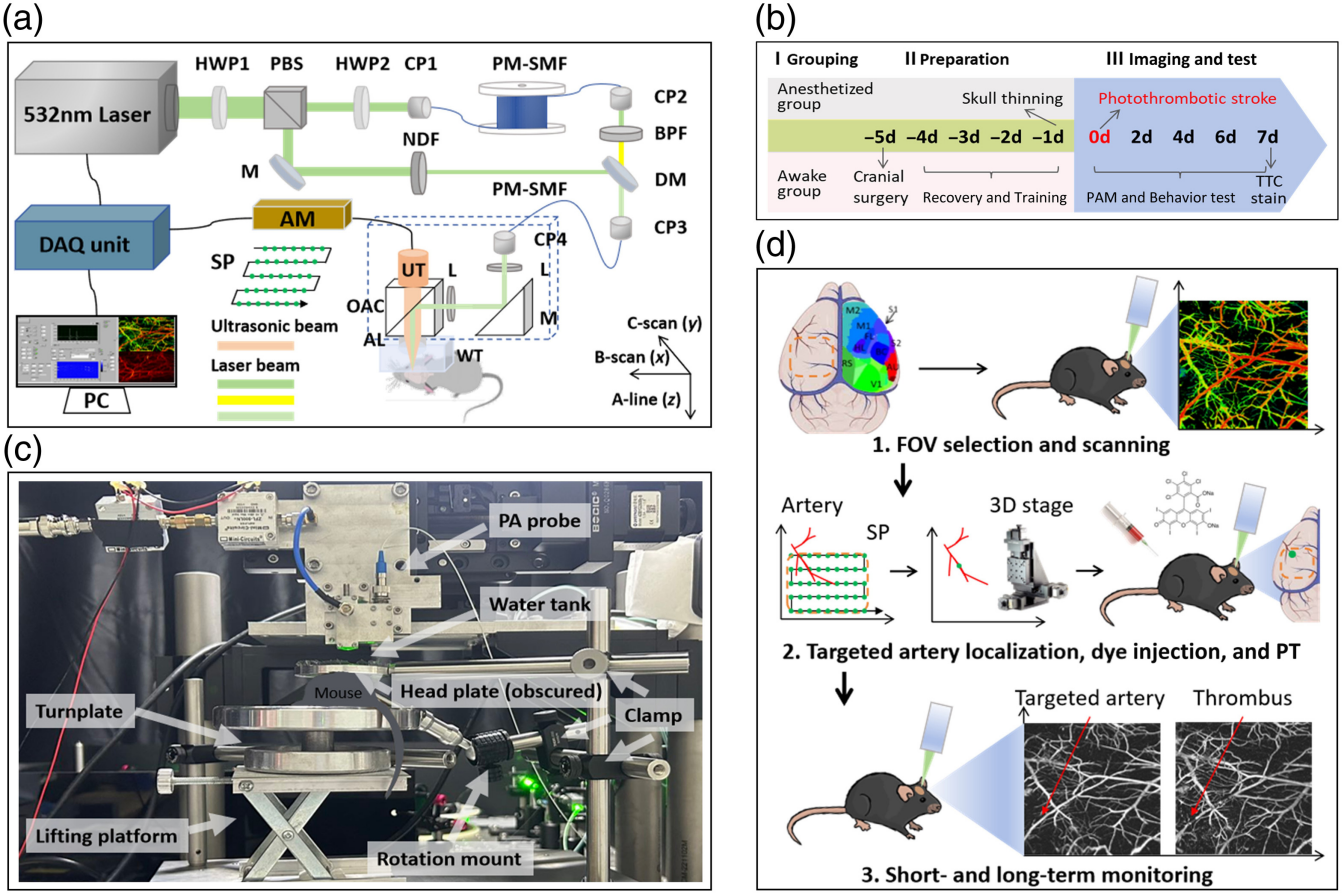
Experimental design and system setup. (a) Schematic design of the custom-built dual-wavelength OR-PAM system, illustrating optical and acoustic pathways. HWP, half-wave plate; PBS, polarizing beam splitter; CP, coupler; PM-SMF, polarization-maintaining single-mode fiber; BPF, band-pass filter; M, mirror; NDF, neutral density filter; DM, dichroic mirror; L, achromatic lens; OAC, optical/acoustic beam combiner; AL, acoustic lens; WT, water tank; UT, ultrasound transducer; AM, amplifier; DAQ, data acquisition unit; PC, personal computer; SP, scanning pathway. (b) Timeline of research including mouse grouping, skull-thinning, head-fixation training, PT induction, PAM imaging, behavioral tests, and pathological examination. (c) Schematic diagram of the head-restrained awake PAM setup. PA probe, photoacoustic probe. (d) Illustration of targeted PT induced in a single distal MCA branch using 532-nm laser illumination guided by PAM. Brain region abbreviations: M1/M2, primary/secondary motor cortex; S1/S2, primary/secondary somatosensory cortex; V1, visual cortex; AU, auditory cortex; RS, retrosplenial area; BC, barrel field; FL and HL, forelimb and hindlimb regions of the primary somatosensory area. The mouse brain atlas image is adapted from Ref. 20.

Figure 1(a) shows the custom-built dual-wavelength OR-PAM system. A 532-nm pulsed laser (VGEN-G-HE-30, Spectra-Physics, Milpitas, California, United States) was divided into two optical paths via a polarizing beam splitter (PBS). One laser beam was coupled into a 35-m polarization-maintaining single-mode fiber (PM-SMF; HB450-SC, Fibercore, Southampton, United Kingdom) to generate a 558-nm wavelength through the stimulated Raman scattering (SRS) effect. A half-wave plate (HWP) was employed to adjust the polarization of the laser beam entering the 35-m PM-SMF to maximize the SRS efficiency. A band-pass filter (BPF, 87-887, Edmund Optics, Barrington, New Jersey, United States) isolated the 558-nm wavelength, and a neutral density filter (NDF) adjusted the 532-nm pulse energy. Both laser beams were subsequently recombined using a dichroic mirror (DM, T550lpxr-UF1, Chroma Technology, Bellows Falls, Vermont, United States) and delivered via a 2-m SMF to the imaging probe.

Within the probe, the output laser beam from the SMF was first collimated and focused by a pair of achromatic lenses (L, AC064-013-A, Thorlabs, Newton, New Jersey, United States) and then reflected by the optical/acoustic combiner (OAC) to illuminate the sample, with pulse energy at the surface of mouse brain of ∼45  nJ per wavelength at a pulse repetition rate (PRR) of 4 kHz. The OAC was fabricated by bonding an aluminum-coated prism to an uncoated prism (32-331 and 32-330, Edmund Optics) using an adhesive (NOA 61, Norland Products, Barrington, New Jersey, United States). The acoustic signals were collected by the acoustic lens (AL, 45-697, Edmund Optics), transmitted through the OAC, and detected by a broadband piezoelectric transducer (50-MHz center frequency, 78%-bandwidth, V214-BC-RM, Olympus, Shinjuku, Japan). Optical and acoustic foci were confocally and coaxially aligned to maximize probe sensitivity. The photoacoustic signals were amplified by 48 dB using a pair of amplifiers (ZFL-500LN, Mini-Circuits, Brooklyn, New York, United States) and digitized at 500 MHz via a data acquisition card (ATS9371, Alazar Technologies, Quebec, Canada). The probe was mounted on a three-axis translational stage (MTS203, Beijing Optical Century Instrument, Beijing, China), and raster-scan over a $3\times 3\;{\mathrm{mm}}^{2}$ field of view (FOV) with a 2.5  μm step size was performed in 6 min, yielding a total fluence of $∼1.44\;J/{\mathrm{cm}}^{2}$ per scan with an average power density of $∼4\;\mathrm{mW}/{\mathrm{cm}}^{2}$. The lateral resolution of the OR-PAM system was 4.7  μm.^21^^,^^22^ To further demonstrate the imaging capabilities of our system, representative PAM and cross-sectional images of the cortical microvasculature are provided in Fig. S1 in the Supplementary Material.

Image reconstruction and quantitative analyses were performed using MATLAB (Version 2018b, MathWorks, Natick, Massachusetts, United States). Structural parameters included arteriovenous diameter, total vessel length (TVL), branch index (BI), and vessel area density (VAD), whereas functional analysis quantified ${\mathrm{sO}}_{2}$ and oxygen extraction fraction (OEF).^23^^–^^25^ A detailed flowchart is illustrated in Fig. S2 in the Supplementary Material. Raw PA amplitude data at each wavelength were first reconstructed into maximum intensity projection (MIP) images and normalized. Median and Gaussian filtering were then applied sequentially to reduce background noise and enhance contrast between vascular structures and surrounding tissue. Vessel segmentation was performed using Otsu’s optimal thresholding on the preprocessed MIP images, followed by morphological operations and short-segment pruning to eliminate nonvessel artifacts. Finally, vessel skeletonization was conducted to extract centerlines and detect branch points for subsequent morphometric quantification.

• TVL: Skeletonized centerlines were extracted and calibrated to physical length (mm).
• BI: Quantified as the number of vascular branch points per FOV.
• VAD: Calculated as the ratio of vessel-occupied pixels to total FOV pixels (%).
• ${\mathrm{sO}}_{2}$: Calculated via spectral unmixing of PA signals at dual wavelengths, based on the optical absorption of oxygenated hemoglobin (${\mathrm{HbO}}_{2}$) and deoxygenated hemoglobin (HbR): ${\mathrm{sO}}_{2}=[{\mathrm{HbO}}_{2}]/([{\mathrm{HbO}}_{2}]+[\mathrm{HbR}])$, where brackets denote concentration.
• OEF: Derived from ${\mathrm{sO}}_{2}$ of feeding arteries ($s_{a}O_{2}$) and draining veins ($s_{v}O_{2}$): $\mathrm{OEF}=(s_{a}O_{2}-s_{v}O_{2})/s_{a}O_{2}$.

### 2.2 Animal Preparation

Male C57BL/6 mice (∼6- to 8-week-old, ∼19- to 23-g weight) were housed four per cage under a 12-h light/dark cycle with free access to food and water. All animal procedures were approved by the Ethics Committee of Guangdong Provincial People’s Hospital (KY2025-1210-01). The experimental timeline for grouping, preparation, imaging, behavioral test, and pathological examination is shown in Fig. 1(b). No animals were reused across OR-PAM imaging, behavioral tests, or pathological examination, and all experimental groups were independent.

Mice in the awake group underwent skull-thinning and head-restraint implantation following established protocols, which were widely used in neurological research, including stroke models.^6^^,^^26^^,^^27^ These minimally invasive approaches preserved intracranial integrity, averted surgery-induced inflammation and vascular tone shifts associated with open craniotomy, and maintained cerebrovascular structure and oxygenation to avoid confounding infarct vulnerability.^12^^,^^28^^,^^29^ Anesthesia was induced with 3% isoflurane (RWD Life Science, Shenzhen, Guangdong, China) and maintained at 1% after loss of the paw-withdrawal reflex. The scalp was depilated, disinfected with 75% ethanol and 10% povidone-iodine, and incised along the midline to expose the skull. The meninges were gently removed with sterile cotton swabs. A dental drill (0.5 mm diamond burr) thinned a $3\times 3\;{\mathrm{mm}}^{2}$ region over the left cortex (2 mm lateral, 1.5 mm posterior to bregma) to ∼200  μm, with saline perfusion to prevent overheating. An M3 brass nut (diameter 3 mm, height 2.4 mm) was affixed using instant adhesive and encapsulated with dental cement (Glass Ionomer Cement Type I, Shanghai New Century Dental, Jiading District, China). Mice were kept at ∼37°C during all the procedure and recovered on a heating pad before being returned to individual cages. The entire procedure lasted for no more than 1 h. Isoflurane had been reported to undergo rapid pulmonary clearance, with cerebrovascular and cerebral metabolic tone returning to pre-anesthesia baseline levels within 1 h after cessation.^30^ During the following 4 days, mice were trained three times daily (45 min/session) with the head-restraint apparatus [Fig. 1(c)] following standard adaptation procedures, and the 4-day recovery window was sufficient for the subsidence of acute surgical stress and restoration of physiological baseline levels.^31^ The head-mount was fixed via a nut-and-clamp system on a turnplate and a lifting platform, which allowed free limb movement and posture adjustment. Sunflower seeds were used as positive reinforcement. PT and all subsequent PAM imaging were performed without anesthesia.

Mice in the anesthetized group received identical skull-thinning surgery 1 day before stroke but without head-restraint implantation or training. The scalp was sutured with 6-0 nylon post-surgery. Accordingly, PT and all subsequent PAM imaging were performed under 1.5% isoflurane anesthesia.

### 2.3 Photothrombotic Stroke Induction

As depicted in Fig. 1(d), photothrombotic stroke induction was performed through three sequential steps. First, a $3\times 3\;{\mathrm{mm}}^{2}$ FOV was initially scanned using OR-PAM to acquire simultaneous vascular structural and ${\mathrm{sO}}_{2}$ maps. This allowed for the identification of distal branches of the MCA within the primary somatosensory cortex, confirmed by ${\mathrm{sO}}_{2}$ distribution and vascular morphology. Second, the three-axis translational stage and stepper motor were controlled to precisely localize the imaging probe to the identified distal MCA branch. Rose Bengal (330,000, Sigma-Aldrich, St. Louis, Missouri, United States) was prepared as a 3  mg/mL solution in sterile saline and injected intraperitoneally at 10  μL/g body weight. After 5 min of systemic circulation, a continuous-wave (CW) 532-nm laser was focused on the targeted single distal MCA branch for 15 min following established protocols.^32^^,^^33^ The laser was operated at a power of ∼20  mW, with an effective beam spot size of ∼40  μm at the cortical surface. This configuration yielded an estimated irradiance of $∼1591\;W/{\mathrm{cm}}^{2}$ at the target, with a total optical dose of 18 J delivered, sufficient for stable thrombus formation while remaining below the threshold for nonspecific thermal tissue damage. Notably, the power and irradiance of the continuous-wave (CW) laser used for PT induction were several orders of magnitude higher than those of the imaging pulsed laser, and imaging parameters remained far below the thresholds for photochemical or thermal damage to cerebral microvasculature.^32^^,^^34^ To ensure laser beam alignment and stability, rigid mechanical immobilization was implemented for all mice. Awake mice were secured with a custom head-restrained apparatus following 4 days of acclimatization to minimize spontaneous movement, whereas anesthetized mice were fixed in a stereotaxic frame. Finally, real-time OR-PAM feedback, including A-line signal attenuation and C-scan imaging, was used to confirm successful occlusion, characterized by diminished vascular signal intensity, vessel disappearance, and reduced ${\mathrm{sO}}_{2}$ in the targeted artery.^34^ Consistent alignment of the FOV before and after PT further ensured that the total optical dose was accurately and exclusively delivered to the targeted branch. Following laser illumination and confirmatory OR-PAM imaging, mice were recovered on a heating pad and returned to cages with ad libitum access to food and water. General condition, grooming, and posture were monitored for 24 h, with subsequent short- and long-term neurovascular changes tracked via OR-PAM.

### 2.4 Behavioral Tests

All behavioral assessments were performed by the same investigator between 8:00 and 10:00 a.m. in a quiet and temperature-controlled environment to minimize variability.^35^ Tests were administered in the following order: open-field, beam-balance, and grip-strength tests.

• Open-field test: Each mouse was placed at a regular corner of a $45\times 45\times 45\;{\mathrm{cm}}^{3}$ blue acrylic arena and tracked for 10 min using a digital camera. Parameters were analyzed with ToxTrac software (v025.1.1).^36^ The arena was sanitized with 70% ethanol and air dried between sessions to avoid odor interference.
• Beam-balance test: Mice were trained for 2 days (three trials/day) to traverse a 1 m long and 10 mm wide beam elevated 1 m above the ground before grouping. The time required to cross the beam was recorded.
• Grip-strength test: Forelimb and hindlimb strength were quantified using a digital grip-strength meter. Mice were allowed to grasp the metal bar, and the peak force during gentle backward pulling was recorded. Each session included three trials, and averages were calculated.

### 2.5 Triphenyltetrazolium Chloride (TTC) Staining

On day 7 post-stroke, mice were euthanized by cervical dislocation following imaging and behavioral tests. Brains were carefully removed, rinsed in ice-cold saline, and coronally sectioned into 2 mm slices using a rodent brain matrice. Sections were incubated in 1% TTC solution (BL1214A, Biosharp, Beijing, China) at 37°C for 20 min in the dark and then fixed in 4% paraformaldehyde (BL539A, Biosharp) for 24 h. Infarct (pale) regions were quantified using ImageJ (Fiji v2.14.0, National Institutes of Health, Bethesda, Maryland, United States), and infarct ratios were calculated as (infarct area / total brain area) × 100%.

### 2.6 Statistical Analysis

**Fig. 2:**
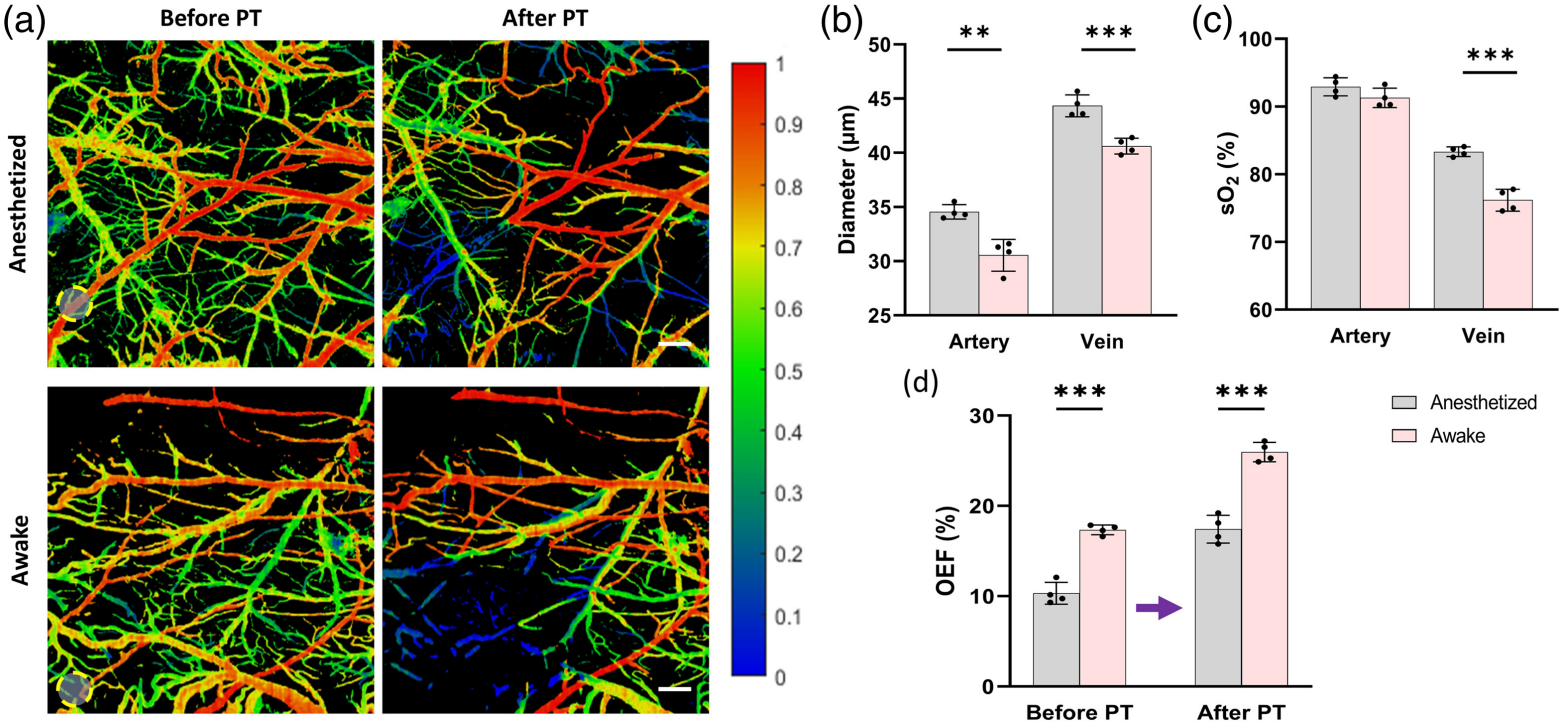
Acute-phase cerebrovascular alterations induced by anesthesia and PT. (a) Representative ${\mathrm{sO}}_{2}$ maps obtained by PAM before (left) and after (right) PT in anesthetized (top) and awake (bottom) mice. Yellow circles indicate the PT site. Scale bar: 300  μm. (b), (c) Quantitative comparison of arteriovenous diameter and ${\mathrm{sO}}_{2}$ between groups induced by anesthesia before PT. (d) Acute changes in OEF before and after PT. Data are presented as mean ± SD (N=4/group). *p<0.05, **p<0.01, ***p<0.001.

**Fig. 3:**
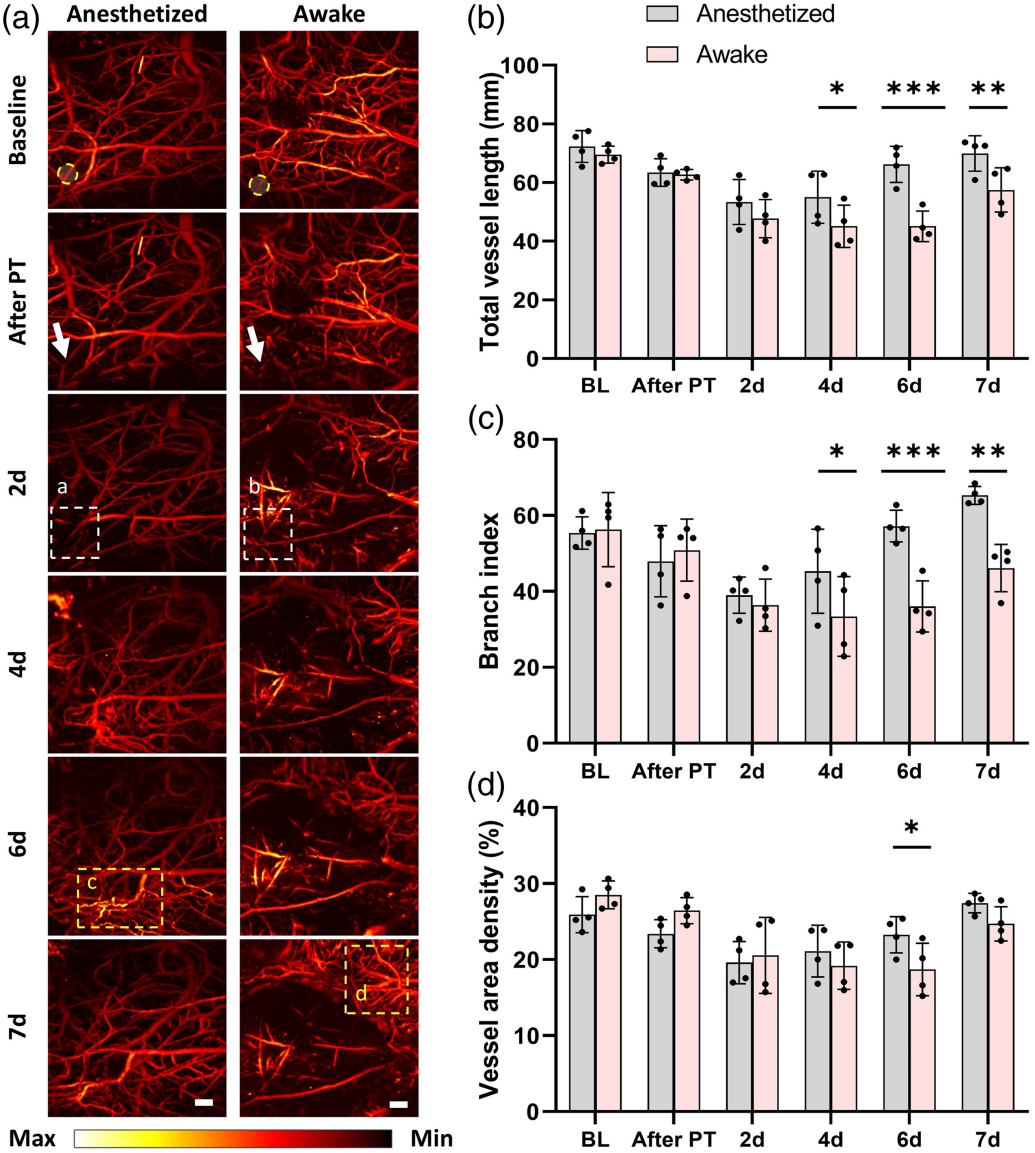
Longitudinal monitoring of vascular remodeling after stroke. (a) Representative PAM images showing microvascular morphology at baseline, after PT, on days 2, 4, 6, and 7 post-stroke in anesthetized (left) and awake (right) mice. Yellow circles indicate the PT site. White arrows point to the affected targeted artery. White rectangles in panels (a) and (b) denote ischemic areas, and yellow rectangles in panels (c) and (d) indicate neovascularization regions. Scale bar: 300  μm. (b)–(d) Longitudinal quantitative comparison of TVL, BI, and VAD between groups. BL, baseline. Data are presented as mean ± SD (N=4/group). *p<0.05, **p<0.01, ***p<0.001.

**Fig. 4:**
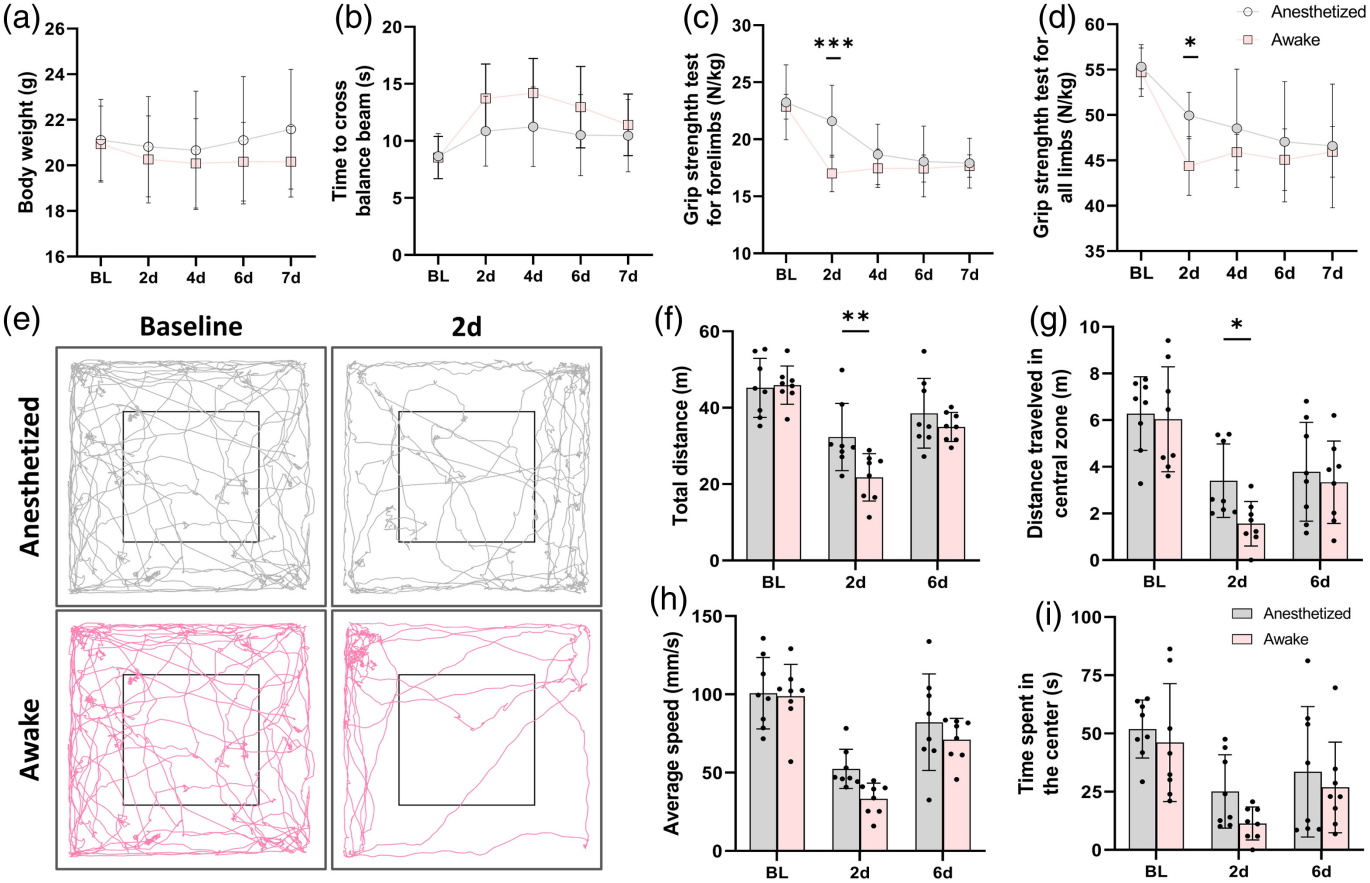
Behavioral performance following photothrombotic stroke. (a) Body weight changes, (b) beam-balance test results, (c) grip strength of forelimbs, and (d) grip strength of all limbs at baseline and on days 2, 4, 6, and 7 post-stroke. (e) Representative open-field trajectories at baseline (left) and on day 2 post-stroke (right) in anesthetized (top) and awake (bottom) mice. (f)–(i) Quantitative analysis of total travelling distance, distance in the central zone, average speed, and residence time in the center. Data are presented as mean ± SD (N=8/group). *p<0.05, **p<0.01, ***p<0.001.

**Fig. 5:**
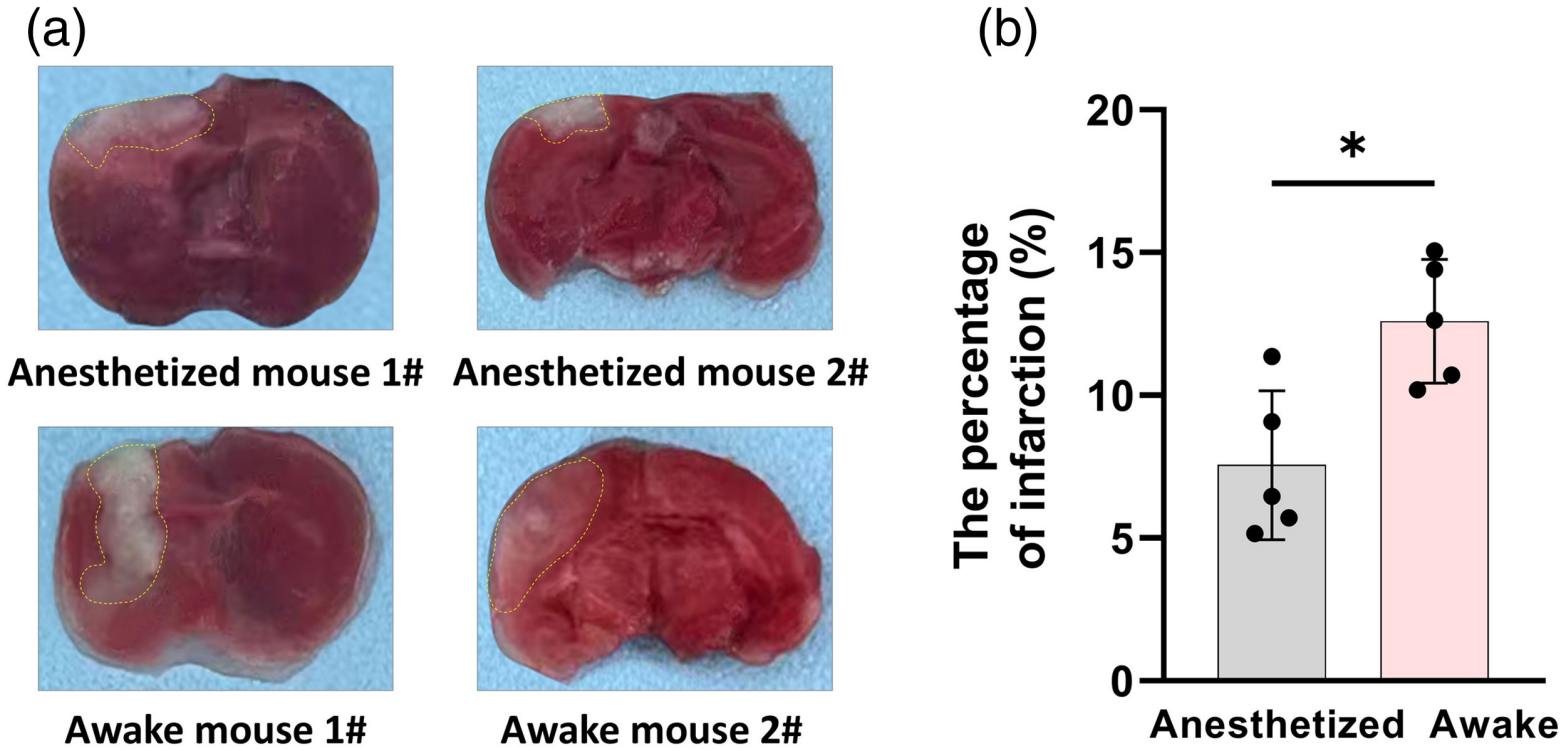
Validation of infarct size using TTC staining. (a) Representative coronal brain sections stained with TTC showing infarct regions (yellow dashed area) in anesthetized (top) and awake (bottom) mice on day 7 post-stroke. (b) Quantitative comparison of infarct ratios between groups. Data are presented as mean ± SD (N=5/group). *p<0.05, **p<0.01, ***p<0.001.

Statistical analyses were performed using GraphPad Prism (Version 9.5.0, GraphPad Software) and R software (Version 4.4.1, R Foundation for Statistical Computing, Vienna, Austria). All statistical tests employed in this study used the individual animal as the independent experimental unit (N), where N represents the number of mice per group. For group comparisons at single time points, an unpaired t-test was employed in Figs. 2(b)–2(c) and 5(b). A two-way analysis of variance (ANOVA) was used to evaluate acute changes in OEF in Fig. 2(d). For longitudinal repeated measurements in morphology and behaviors, linear mixed-effects models were implemented in Figs. 3(b)–3(d), 4(a)–4(d), and 4(f)–4(i) using the “lme4,” “lmerTest,” and “emmeans” packages in R. Standardized effect sizes (Cohen’s d) with 95% confidence intervals (CI) were calculated via the “effectsize” package. All graphical visualizations were generated using GraphPad Prism. Data are presented as mean ± standard deviation (SD). Statistical significance was defined as p<0.05 (p<0.05, *; p<0.01, **; p<0.001, ***).

## 3 Results

### 3.1 Acute-Phase Cerebrovascular Alterations in Anesthetized and Awake Mice after PT

Cerebrovascular parameters measured before PT differed significantly between groups, reflecting the acute physiological effects of anesthesia [Fig. 2(a), left]. Anesthetized mice had larger arteriovenous diameters [Fig. 2(b), artery: 34.55  μm versus 30.55  μm; vein: 44.34  μm versus 40.62  μm] and higher venous ${\mathrm{sO}}_{2}$ [Fig. 2(c), right, ${\mathrm{sO}}_{2}$: 83.33% versus 76.18%] compared with awake mice, whereas arterial ${\mathrm{sO}}_{2}$ showed no significant difference [Fig. 2(c), left, 92.93% versus 91.28%]. The OEF before PT was notably lower in anesthetized mice than that in awake mice [Fig. 2(d), left, 10.32% versus 17.35%].

Following PT, awake mice exhibited more severe reduction in mean ${\mathrm{sO}}_{2}$ and more pronounced elevation in OEF compared with the anesthetized mice [Fig. 2(d), right, 17.43% versus 25.96%]. Representative ${\mathrm{sO}}_{2}$ maps in Fig. 2(a) showed that vascular signal attenuation in the peri-lesional area was more prominent in awake mice, indicating exacerbated oxygenation deficits associated with unmodulated cerebral metabolic demand under awake conditions.

### 3.2 Longitudinal Monitoring of Cerebrovascular Structural Remodeling after PT

We continuously monitored cortical vascular structure for 7 days following PT and observed distinct recovery trajectories between the two groups (Fig. 3). As shown in the representative time-series images [Fig. 3(a)], the awake group exhibited marked vascular rarefaction and branch fragmentation on day 2, whereas the anesthetized group preserved a more integrated vascular network. By day 7, vascular density in awake mice had not fully recovered to baseline or to the level of the anesthetized group.

Quantitative metrics, including TVL, BI, and VAD, were comparable at baseline [Fig. 3(b)–3(d)]. However, all structural indicators in the awake group showed significantly greater reductions in TVL, BI, and VD than those in the anesthetized group. Furthermore, during recovery, anesthetized mice displayed a progressive restoration beginning on day 4, with most parameters approaching baseline by day 7. By contrast, recovery in awake mice was delayed and incomplete, indicating persistent vascular dysfunction and slower angiogenic remodeling. Detailed statistical results are provided in Table S1 in the Supplementary Material.

### 3.3 Behavioral Outcomes Following Photothrombotic Stroke

To correlate vascular alterations with functional outcomes, behavioral tests were assessed longitudinally (Fig. 4). No significant differences in body weight were observed between groups throughout the experiment [Fig. 4(a)]. The beam-balance test revealed that awake mice exhibited prolonged beam-crossing times on day 2 post-stroke and failed to attain the performance levels of the anesthetized group by day 7, although without significant differences [Fig. 4(b)]. Forelimb and overall grip strength in awake mice were significantly reduced on day 2 and recovered partially by day 7 [Figs. 4(c)–4(d)]. Open-field trajectories [Fig. 4(e)] typically demonstrated that awake mice primarily remained near the arena periphery at day 2, indicating decreased locomotor activity. Quantitatively, total travel distance and central zone distance were significantly lower in the awake group on day 2 [Figs. 4(f)–4(g)], although average speed and residence time in the central zone did not differ significantly between groups [Figs. 4(h)–4(i)]. Detailed statistical results are provided in Tables S2 and S3 in the Supplementary Material.

Together, these results confirmed that awake mice experienced more severe and persistent motor and exploratory deficits consistent with impaired neurovascular recovery.

### 3.4 Pathological Validation of Infarct Volume

Histological analysis using TTC staining on day 7 corroborated the imaging and behavioral findings (Fig. 5). Representative coronal brain sections revealed markedly larger infarcted regions in awake mice than in anesthetized counterparts [Fig. 5(a)]. Quantitative comparison confirmed a significantly higher infarct volume ratio in the awake group [Fig. 5(b)], consistent with their more pronounced vascular and behavioral impairments.

## 4 Discussion

The translational gap between preclinical stroke research and clinical outcomes has long been attributed to two interconnected limitations: the confounding effects of anesthesia on cerebrovascular pathophysiology and the technical constraints of conventional imaging modalities in capturing concurrent structural and functional microvascular dynamics. The present study addresses these bottlenecks by integrating functional OR-PAM with a head-restrained awake mouse model, enabling targeted PT of a single vessel and longitudinal monitoring free from anesthetic interference. Experimental results show that the exacerbated vascular degeneration in awake mice likely reflects the absence of anesthesia-induced vasodilation and metabolic suppression. Our findings not only validate the profound neuroprotective role of isoflurane but also establish a more physiologically authentic framework for decoding ischemic stroke pathophysiology.

One of the innovations of this study lies in its methodological refinement of the PT model. Conventional PT often suffers from poor reproducibility due to nontargeted illumination and inconsistent infarct formation. Salman et al.^37^ and Clark et al.^38^ improved localization through artery-targeted PT approaches. However, their approaches lacked the capability for simultaneous oxygen monitoring with high resolution. In the study, OR-PAM was first raster scanned in the FOV to identify the distal MCA branch, and then, the laser spot was precisely localized over the targeted artery by controlling the translational stage and the stepper motor. Furthermore, the occlusion could be timely confirmed and longitudinally monitored. Therefore, our OR-PAM-guided PT strategy enables precise artery and venous identification, real-time confirmation of occlusion, and high spatial reproducibility, which is beneficial for the research on specific mouse neocortex function and blood supply area. These advantages collectively enhance both the reliability and interpretive power of preclinical stroke models.

Traditional optical methods have provided valuable insights into cerebrovascular dynamics but remain constrained by single-parameter measurements. For example, Balbi et al.^12^ employed LSCI to assess CBF in awake mice but failed to quantify oxygenation or vascular morphology. Liang et al.^39^ demonstrated OCT’s strength in post-stroke vascular imaging to illustrate the association between age and stroke but lacked functional data. OR-PAM resolves these deficiencies by exploiting hemoglobin’s endogenous absorption contrast, enabling noninvasive and concurrent visualization of vascular structure and function. Previous studies successfully applied OR-PAM for cortex-wide hemodynamic mapping in awake rodents.^6^^,^^28^ Our results regarding arteriovenous diameters, ${\mathrm{sO}}_{2}$, and OEF induced by anesthesia before PT between awake and anesthetized mice were similar to theirs. Furthermore, OEF was shown to have significantly deteriorated in awake mice after PT, which was consistent with the previous results.^7^ OEF serves as a marker of regional oxygen extraction efficiency, reflecting how effectively brain tissue takes up oxygen from the circulating blood.^40^ As a relative index of tissue oxygen demand and utilization, OEF has been widely adopted in neurovascular and stroke research to characterize the severity of ischemic challenge. Notably, advanced studies have extended this framework by simultaneously measuring CBF and cerebral metabolic rate of oxygen (${\mathrm{CMRO}}_{2}$), which is a gold-standard metric for quantifying the absolute amount of oxygen consumed by brain tissue per unit time.^19^^,^^41^ ${\mathrm{CMRO}}_{2}$ integrates the combined contributions of perfusion supply (delivered via CBF) and tissue oxygen extraction (reflected by OEF), embodying the critical coupling between blood-borne oxygen delivery and metabolic demand at the tissue level. Under relatively stable hemodynamic conditions, OEF and ${\mathrm{CMRO}}_{2}$ often change in a consistent manner, supporting the value of OEF as a functionally relevant surrogate.^28^^,^^42^ However, both do not always follow identical trends as shifts in perfusion can independently alter oxygen extraction without a corresponding change in actual metabolic consumption.^43^^,^^44^ Building on these advances, the present study integrates OR-PAM with targeted PT induction, allowing real-time vascular structure and function correlation, and overcoming the inherent constraints of traditional optical modalities.

Anesthetic interference remains a fundamental confounder in cerebrovascular research. Isoflurane has been reported to induce cerebral vasodilation, depress neuronal activity, and alter neurovascular coupling.^45^ Lyons et al.^46^ demonstrated distinct oxygenation gradients between awake and anesthetized brains, whereas Sciortino et al.^6^ further confirmed baseline disparities in arteriovenous ${\mathrm{sO}}_{2}$ gradients. Recent technical advances, including transparent cranial windows^47^ and head-mounted OR-PAM systems,^48^ have enabled stable awake-state imaging, supporting the transition toward nonanesthetic paradigms. Consistent with these findings, our results highlight that awake mice models reveal the true magnitude of ischemic injury and recovery dynamics otherwise masked under anesthetic neuroprotection.

The behavioral outcomes in this study establish a direct link between vascular and neurological alterations. Previous reports have emphasized this translational relevance. Ruan and Yao^35^ underscored the need for behavioral-vascular correlation in stroke models, and Kalantari et al.^49^ characterized two distinct recovery trajectories following cortical stroke without direct vascular mapping. Here, we found that the changing trends of vascular structural and functional parameters (including the temporal nodes of maximal impairment and recovery) differed from those of behavioral metrics, although the awake group generally exhibited more severe impairment and slower recovery than the anesthetized group. The combination of behavioral metrics and OR-PAM-derived parameters (e.g., VAD and ${\mathrm{sO}}_{2}$ alteration) provides a mechanistic bridge between microvascular remodeling and neurological function, strengthening the physiological interpretability of the model. Besides, a deeper investigation of targeted cortical brain region ischemia and behavior deficits is feasible by combining OR-PAM and PT induction. We integrated TTC staining, a gold-standard histological assay for infarct volume quantification, into our multimodal assessment. OR-PAM enables longitudinal and high-resolution monitoring of microvascular remodeling in the superficial cortex, which is constrained by the thinned-skull preparation. By contrast, TTC staining evaluates the total infarct volume throughout the entire brain parenchyma. Importantly, photothrombotic lesions form a columnar pattern that extends through the cortical depth.^34^^,^^50^ This anatomical feature ensures that superficial microvascular alterations detected by OR-PAM accurately reflect the overall ischemic severity, bridging early microscale hemodynamic changes to the brain’s terminal metabolic outcome.

Despite its advantages, several limitations in the study should be addressed for further exploration. Inevitably, the awake cohort was exposed to brief anesthesia for skull-thinning and head-restraint implantation, and additional head-fixation acclimation was required, introducing a slight potential for experimental confounds. It would be rigorous to monitor blood corticosterone levels or perform an MRI to exclude the confounds before PT induction and subsequent imaging.^51^ However, this scenario is unlikely to affect our core findings as both groups underwent the same short anesthetic exposure for surgical preparation, and no significant intergroup differences were observed in baseline cerebrovascular morphology or behavioral parameters. Moreover, we still detected robust disparities in infarct volumes and post-stroke outcomes. Second, the limited FOV restricts assessment to local cortical regions and precludes cortex-wide analysis of network-level neurovascular coordination. Expanding the imaging field with a whole cranial window or incorporating wide-field photoacoustic computed tomography could address this limitation.^52^ Third, we did not directly measure CBF or ${\mathrm{CMRO}}_{2}$ in the present study. Without these key hemodynamic and metabolic measures, we cannot fully disentangle the relative contributions of perfusion supply and tissue metabolic demand to the observed changes in OEF. This also prevents us from establishing a complete picture of the oxygen supply, extraction, and consumption cascade, which limits the mechanistic depth of our interpretation regarding the metabolic state of the ischemic tissue. Integration of multiwavelength or photoacoustic Doppler techniques would enable a comprehensive assessment of these metrics.^53^^,^^54^ Finally, integrating electrophysiological or molecular imaging modalities could provide deeper mechanistic insights and further enhance the translational relevance of this platform.

## 5 Conclusion

This study establishes a single OR-PAM-based framework integrated with a head-restrained awake mouse model for simultaneous targeted photothrombotic stroke induction and longitudinal monitoring compared with traditional approaches. Furthermore, it noninvasively and quantitatively delineates anesthetic confounding impact on cerebrovascular and ${\mathrm{sO}}_{2}$ dynamics, demonstrating that awake-state imaging captures authentic ischemic pathology masked under anesthesia. The functional OR-PAM guided-PT on awake rodent models offers a translationally relevant tool for mechanistic stroke research and potential optimization of clinical imaging protocols.

## Supplementary Material

10.1117/1.NPh.13.2.025007.s01

## Data Availability

Code and data will be made available upon reasonable request.
