# Supplementary material for: Inducing and monitoring photothrombotic stroke in anesthetic neuroprotection-free mice using functional photoacoustic microscopy
Source: Neurophotonics. 2026 Apr 15;13(2):025007. doi: 10.1117/1.NPh.13.2.025007 (PMC13082355; doi:10.1117/1.NPh.13.2.025007)
Supplement: Supplementary file 1 [file NPh_013_025007_SD001.pdf]

Supplementary Information for

Inducing and Monitoring Photothrombotic Stroke in Anesthetic Neuroprotection-Free Mice Using Functional Photoacoustic Microscopy

Congsen Li,[a] Kexin Chen,[a] HaiPing Cai,[b] Xiaobin Hong,[a,*] and Jiangbo Chen[a,*]

[a]School of Mechanical & Automotive Engineering, South China University of Technology, Guangzhou, Guangdong, PR China

[b]Department of Neurosurgery, Guangdong Provincial People's Hospital (Guangdong Academy of Medical Sciences), Southern Medical University, Guangzhou, Guangdong, PR China

*Address all correspondence to Xiaobin Hong, scut_hongxiaobin@126.com; Jiangbo Chen, cjiangbo@scut.edu.cn

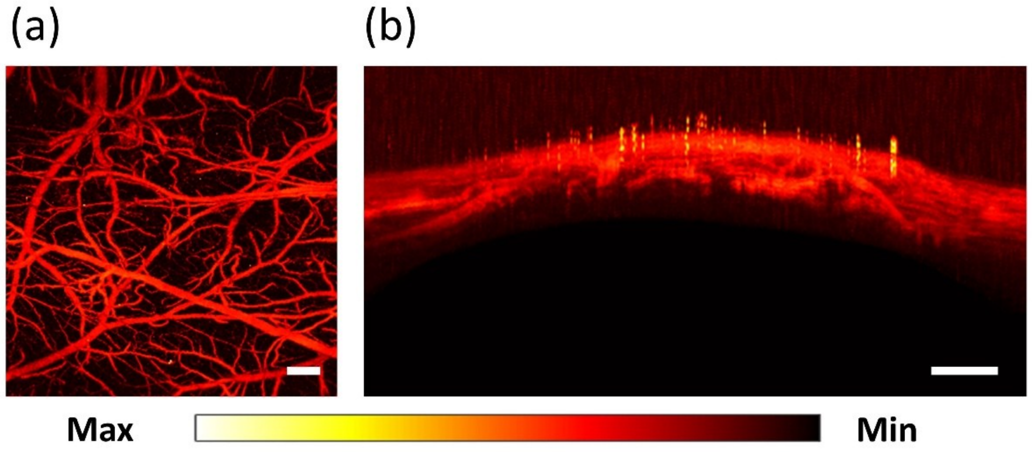

Figure S1. PAM image (a) and its corresponding cross-sectional image (b) of cortical microvasculature. Scale bar: 300 μm.

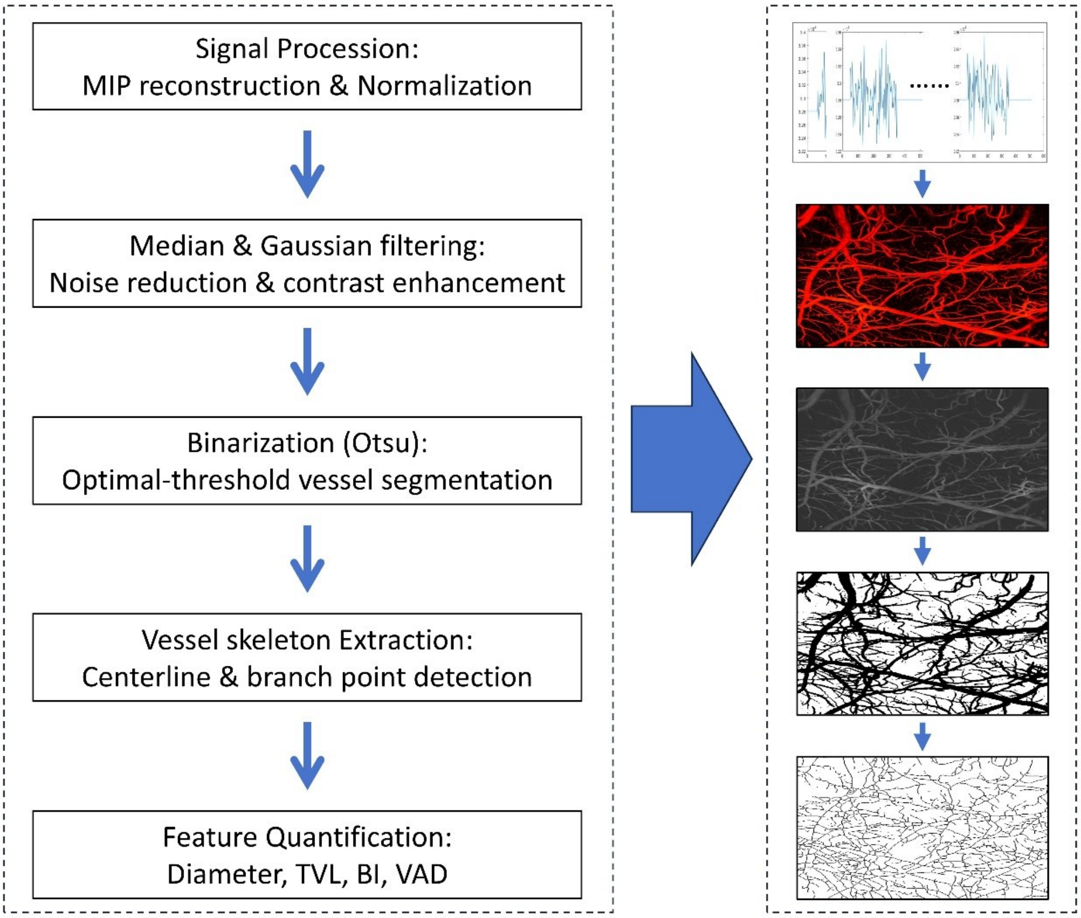

Figure S2. The flowchart of the vascular extraction algorithm. MIP, maximum intensity projection; TVL, total vessel length; BI, branch index; VAD, vessel area density.

Table S1. Effect sizes with confidence intervals corresponding to Figs. 3(b)-3(d)

Awake vs. Anesthetized	Total vessel length		Branch index		Vessel area density	
	Mean difference	Cohen's d	Mean difference	Cohen's d	Mean difference	Cohen's d
**BL**	-2.73 [-11.58, 6.13]	0.48 [-1.08, 2.04]	0.89 [-10.1, 11.89]	-0.15 [-1.95, 1.66]	2.61 [-1.62, 6.85]	-1.4 [-3.7, 0.9]
**After PT**	-0.74 [-9.6, 8.12]	0.13 [-1.43, 1.69]	2.94 [-8.06, 13.94]	-0.48 [-2.3, 1.33]	3.04 [-1.19, 7.28]	-1.63 [-3.94, 0.68]
**2d**	-5.65 [-14.51, 3.21]	0.99 [-0.58, 2.57]	-2.62 [-13.62, 8.38]	0.43 [-1.38, 2.24]	0.95 [-3.28, 5.19]	-0.51 [-2.79, 1.77]
**4d**	-9.91 [-18.76, -1.05]	1.74 [0.13, 3.36]	-11.91 [-22.91, -0.91]	1.96 [0.08, 3.83]	-1.94 [-6.17, 2.3]	1.04 [-1.25, 3.33]
**6d**	-21.1 [-29.95, -12.24]	3.71 [1.9, 5.52]	-21.18 [-32.18, -10.18]	3.48 [1.47, 5.49]	-4.56 [-8.79, -0.32]	2.45 [0.09, 4.81]
**7d**	-12.4 [-21.26, -3.54]	2.18 [0.53, 3.83]	-19.16 [-30.16, -8.16]	3.15 [1.18, 5.12]	-2.73 [-6.96, 1.51]	1.46 [-0.84, 3.77]

All data are presented as mean or Cohen's d ± 95% confidence intervals (N=4/group). Red fonts indicate statistically significant difference.

Table S2. Effect sizes with confidence intervals corresponding to Figs. 4(a)-4(d)

Awake vs. Anesthetized	Body weight		Time to cross balance beam		Grip strength test for forelimbs		Grip strength test for all limbs	
	Mean difference	Cohen's d	Mean difference	Cohen's d	Mean difference	Cohen's d	Mean difference	Cohen's d
**BL**	-0.18 [-2.4, 2.05]	0.25 [-2.88, 3.38]	-0.12 [-3.12, 2.87]	0.05 [-1.16, 1.27]	-0.39 [-2.68, 1.91]	0.24 [-1.17, 1.64]	-0.6 [-5.02, 3.82]	0.22 [-1.42, 1.87]
**2d**	-0.56 [-2.79, 1.66]	0.79 [-2.34, 3.92]	2.87 [-0.12, 5.86]	-1.16 [-2.4, 0.07]	-4.59 [-6.88, -2.29]	2.81 [1.32, 4.3]	-5.55 [-9.97, -1.13]	2.07 [0.38, 3.75]
**4d**	-0.58 [-2.8, 1.65]	0.81 [-2.32, 3.94]	2.96 [-0.03, 5.95]	-1.2 [-2.43, 0.03]	-1.21 [-3.51, 1.08]	0.74 [-0.67, 2.15]	-2.64 [-7.06, 1.78]	0.98 [-0.67, 2.64]
**6d**	-0.94 [-3.16, 1.29]	1.32 [-1.82, 4.46]	2.44 [-0.55, 5.43]	-0.99 [-2.22, 0.24]	-0.61 [-2.91, 1.68]	0.38 [-1.03, 1.78]	-1.98 [-6.4, 2.45]	0.74 [-0.92, 2.39]
**7d**	-1.23 [-3.45, 1]	1.72 [-1.42, 4.87]	1 [-1.99, 3.99]	-0.41 [-1.62, 0.81]	-0.25 [-2.54, 2.04]	0.15 [-1.25, 1.56]	-0.64 [-5.06, 3.78]	0.24 [-1.41, 1.89]

All data are presented as mean or Cohen's d ± 95% confidence intervals (N=8/group). Red fonts indicate statistically significant difference.

Table S3. Effect sizes with confidence intervals corresponding to Fig. 4(f)-4(i)

Awake vs. Anesthetized	Total distance		Distance traveled in central zone		Average speed		Time spent in the center	
	Mean difference	Cohen's d	Mean difference	Cohen's d	Mean difference	Cohen's d	Mean difference	Cohen's d
**BL**	0.71 [-6.49, 7.91]	-0.14 [-1.51, 1.24]	-0.24 [-2.03, 1.55]	0.18 [-1.15, 1.51]	-1.94 [-22.01, 18.13]	0.13 [-1.18, 1.43]	-5.82 [-25.54, 13.9]	0.37 [-0.9, 1.64]
**2d**	-10.56 [-17.75, -3.36]	2.01 [0.57, 3.46]	-1.85 [-3.64, -0.05]	1.37 [0, 2.74]	-19.16 [-39.23, 0.91]	1.25 [-0.09, 2.58]	-13.75 [-33.47, 5.97]	0.88 [-0.4, 2.17]
**6d**	-3.56 [-10.76, 3.63]	0.68 [-0.7, 2.06]	-0.45 [-2.24, 1.34]	0.34 [-1, 1.67]	-11.16 [-31.23, 8.91]	0.73 [-0.59, 2.04]	-6.67 [-26.39, 13.05]	0.43 [-0.84, 1.7]

All data are presented as mean or Cohen's d ± 95% confidence intervals (N=8/group). Red fonts indicate statistically significant difference.
